# Stearic Acid-Modified Calcium Sulfate Whiskers as a Functional Filler for Rubber Enhancement

**DOI:** 10.3390/ma18184355

**Published:** 2025-09-18

**Authors:** Guoying Yan, Peiyang Shi, Linlin Guan, Mengting Liang, Chengjun Liu

**Affiliations:** 1 Key Laboratory for Ecological Metallurgy of Multimetallic Ores, Ministry of Education, Northeastern University, Shenyang 110819, China; kyykykfb@163.com (G.Y.);; 2 School of Metallurgy, Northeastern University, Shenyang 110819, China; 3 Baosteel Group Mine Research Institute, Baotou 014000, China

**Keywords:** calcium sulfate whisker, surface modification, stearic acid, rubber

## Abstract

Calcium sulfate whiskers (CSWs) are fibrous crystals with uniform cross-section, well-defined morphology, and dense structure. Due to their low toxicity and low cost, CSWs have wide applications as additives in composite materials. In this work, CSWs prepared from desulfurized gypsum were used as raw materials. The mechanism of stearic acid (SA) surface modification of CSWs was investigated, and the influence of SA-modified CSWs on the mechanical properties of rubber was evaluated. Results show that SA effectively modifies CSW surfaces through a synergistic mechanism involving chemical bonding and physical adsorption. At lower SA concentrations, surface modification is primarily governed by chemical bonding, whereas physical adsorption becomes increasingly dominant at higher SA concentrations. Consequently, both the activation index and contact angle of modified CSWs initially increase but then decrease with rising SA content, peaking at a 4 wt.% SA dosage. At this optimal concentration, maximum values of 0.636 (activation index) and 110° (contact angle) were achieved. Furthermore, both unmodified and modified CSWs could improve the hardness, tensile strength, and elongation at break of the rubber. The optimal performance was achieved with 4 wt.% SA-modified CSWs, resulting in a hardness of 67°, a tensile strength of 21.92 MPa, and an elongation at break of 619%.

## 1. Introduction

Although global reserves of natural gypsum are abundant, their distribution is highly uneven, and the availability of high-quality natural gypsum is limited. Meanwhile, large quantities of industrial by-product gypsum are generated annually from industries such as flue gas desulfurization (FGD), chemical manufacturing, and environmental treatment. To conserve natural gypsum resources and mitigate the environmental impact of by-product gypsum accumulation, significant research has focused on the utilization of industrial by-product gypsum [1,2,3,4].

Desulfurized gypsum, with its chemical similarity to natural gypsum, is considered as a promising alternative. Utilizing desulfurized gypsum not only conserves natural resources and prevents ecological damage but also facilitates waste valorization, reduces land occupation, and promotes environmental sustainability [5,6,7,8]. In China, the annual production of industrial by-product gypsum exceeds 200 million tons, with over 80 million tons coming from FGD alone. However, its application is currently limited mainly to the construction sector [9,10,11], which has a narrow market capacity. Therefore, an urgent task is to expand the application scope of by-product gypsum. One promising approach is the fabrication of CSWs from desulfurized gypsum. Although the synthesis of CSWs has been reported previously [12,13,14,15], their application in rubber remains underexplored.

Previous studies have demonstrated that whiskers can significantly enhance the properties of rubber composites. For example, Ji et al. [16] prepared carbon nanotube whisker@n-Al_2_O_3_ hybrids via electrostatic self-assembly. When incorporated into silicone rubber, these hybrids improved both thermal conductivity and electrical insulation. Similarly, Silva et al. [17] showed that incorporating carbon nanotube whiskers and bio-glass particles into natural rubber fiber mats enhanced their mechanical properties without compromising thermal stability. Wu et al. [18] developed carbon black/natural rubber nanocomposites with a 3D hierarchical conductive structure by introducing cellulose nanowhiskers, which enhanced sensing capabilities and electrical conductivity. These studies highlight the reinforcing potential of inorganic whiskers in polymer matrices. As ultra-fine short fibers, CSWs are expected to have similar reinforcement effects in rubber, while offering advantages such as lower cost and better utilization of industrial gypsum waste. Their successful application could contribute to both performance enhancement and environmental sustainability. Recent work further supports this potential. Chen et al. [19] found that adding modified CSWs significantly improved the mechanical strength and solvent resistance of silicone rubber, reduced the glass transition temperature, and strengthened interfacial interactions. Li et al. [20] observed uniform dispersion of CSWs in resin matrices, resulting in improved storage modulus, thermal stability, and wear resistance.

Despite these promising findings, the application of modified CSWs in polymer systems remains limited. Therefore, improving the compatibility and reinforcing efficiency of CSWs in rubber is essential for the high-value utilization of industrial by-product gypsum. In this study, we employ a solvent-assisted method to explore the effect of SA concentration on the surface modification of CSWs. The modified whiskers are incorporated into rubber composites, which are then characterized by FT-IR, TEM, and mechanical testing. The relationship between whisker surface modification and the enhancement of rubber properties is systematically investigated, providing both theoretical insights and technical guidance for the application of CSWs in polymer materials.

## 2. Experimental

### 2.1. Preparation of SA-Modified CSWs

CSWs were prepared using desulfurization gypsum from Baotou Iron and Steel (Group) Co., Ltd. (Baotou, China) as shown in our previous study [21]. The CSWs were further modified with SA. First, SA solutions with concentrations of 2 wt.%, 3 wt.%, 4 wt.%, 5 wt.%, and 6 wt.% were prepared in absolute ethanol (Sinopharm Chemical Reagent Co., Ltd., Shanghai, China). The solutions were then heated to 80 °C under continuous stirring. Subsequently, 10 g of CSWs were added, and the mixture was maintained at 80 °C with stirring for 0.5 h. Finally, the modified whiskers were collected by filtration and dried at 50 °C for 1 h.

### 2.2. Preparation of Whisker/Rubber Composites

The whisker/rubber composite was prepared using a formulation consisting of 100 g of natural rubber, 6 g of zinc oxide, 1.5 g of SA, 2.6 g of sulfur, 0.7 g of accelerant MBT (2-mercaptobenzothiazole), 2.0 g of antioxidant ODA (octylated diphenylamine), 1.5 g of antioxidant BHT (butylated hydroxytoluene), 1.5 g of antioxidant TBP (tris(nonylphenyl) phosphite), 48 g of carbon black, 6 g of process oil, 4 g of coumarone resin, and 20 g of calcium carbonate as the initial filler. All chemicals used were analytical-grade reagents, purchased from Sinopharm Chemical Reagent Co., Ltd., Shanghai, China.

In this study, SA-modified CSWs were incorporated as an additional filler at a specified ratio relative to the natural rubber (e.g., 10 wt.% corresponds to adding 10 g of CSWs). All components were mixed into the rubber using a two-roll mill, followed by the addition of sulfur and mixing until homogeneous. The compound was then vulcanized at 148 °C for 10 min under pressure in a platen press, yielding the whisker/rubber composites.

### 2.3. Characterization

(1) Scanning electron microscopy (SEM): SEM images were obtained on a Shimadzu SSX-550 instrument (Kyoto, Japan).
(2) Fourier transform infrared spectroscopy (FT-IR): FT-IR spectra were obtained on a Nicolet 60SXB spectrometer (Waltham, MA, USA) with 4 cm^−1^ resolution, employing the KBr-pellet method.
(3) Activation index: The activation index, defined as the mass fraction of floating modified whiskers, was determined as follows. 1.0 g of SA-modified CSWs was dispersed in 200 mL of deionized water. The dispersion was stirred for 0.5 h and allowed to settle for 2 h until distinct phase separation occurred. The precipitates were then filtered, dried, and weighed. The activation index (*H*) was calculated using the formula: *H* = [(Total Mass − Precipitate Mass)/Total Mass] × 100%.
(4) Contact angle: Contact angles were measured using a Dataphysics OCA20 instrument (Filderstadt, Germany). The powder samples were compressed into smooth and solid cylindrical tablets (φ4.5–6 mm) using a compression molding method prior to measurements. The values reported represent the average of three replicate measurements per sample.
(5) Mechanical properties: Shore A hardness was measured according to GB/T 531-1999 [22] standard using an LX-A durometer. Tensile strength and elongation at break were determined following GB/T 528-1998 [23] standard using a DXLL-3000 electronic tensile tester, Leqing, China. The CSW/rubber composite samples were shaped into a dumbbell for testing.

## 3. Results and Discussion

**Figure 1:**
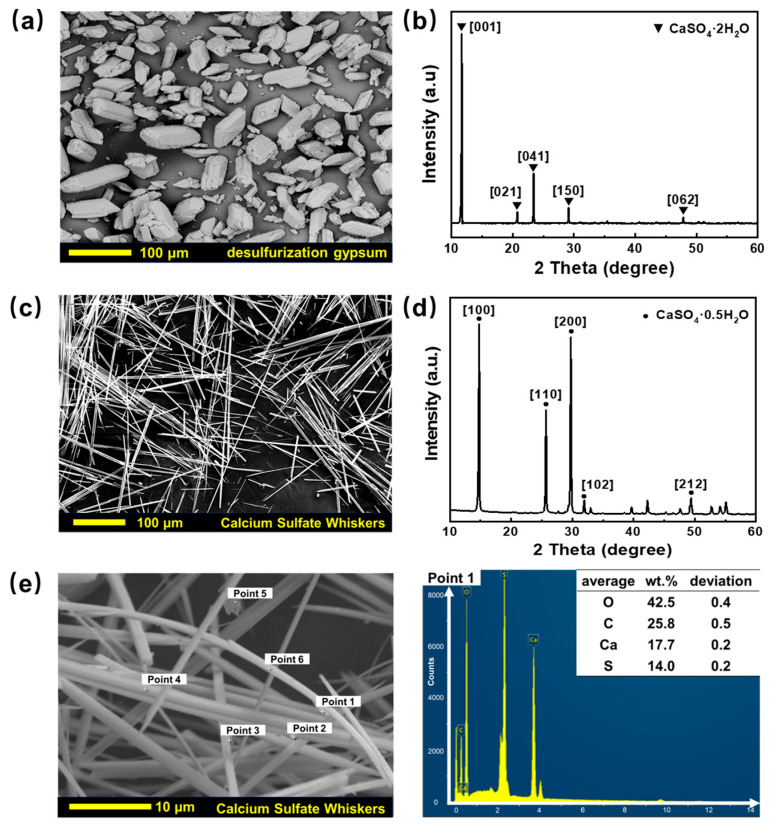
Physical properties of desulfurization gypsum and CSWs prepared from desulfurization gypsum. (**a**) SEM image of desulfurization gypsum; (**b**) XRD pattern of desulfurization gypsum; (**c**) SEM image of CSWs; (**d**) XRD pattern of CSWs; (**e**) EDS point-scanning of CSWs (the inserted table shows the average elemental composition from points 1 to 5).

Figure 1a presents the morphology of the desulfurization gypsum from the Baotou Iron and Steel Group. The raw desulfurization gypsum has an average particle size of ~35 μm and exhibits a variety of morphologies, including short columns, rhomboids, flakes, and spheres. The XRD pattern (Figure 1b) indicates that the main phase is calcium sulfate dihydrate (CaSO_4_·2H_2_O). After hydrothermal treatment, these mixed-phase dihydrate particles were transformed into hemihydrate CSWs with a uniform morphology. The whiskers exhibit an average aspect ratio of 150–200 μm and a mean diameter of 1.5 μm (Figure 1c). The XRD pattern (Figure 1d) indicates that the main phase is calcium sulfate hemihydrate (CaSO_4_·0.5H_2_O). EDS pattern further indicates the composition of calcium sulfate hemihydrate.

**Figure 2:**
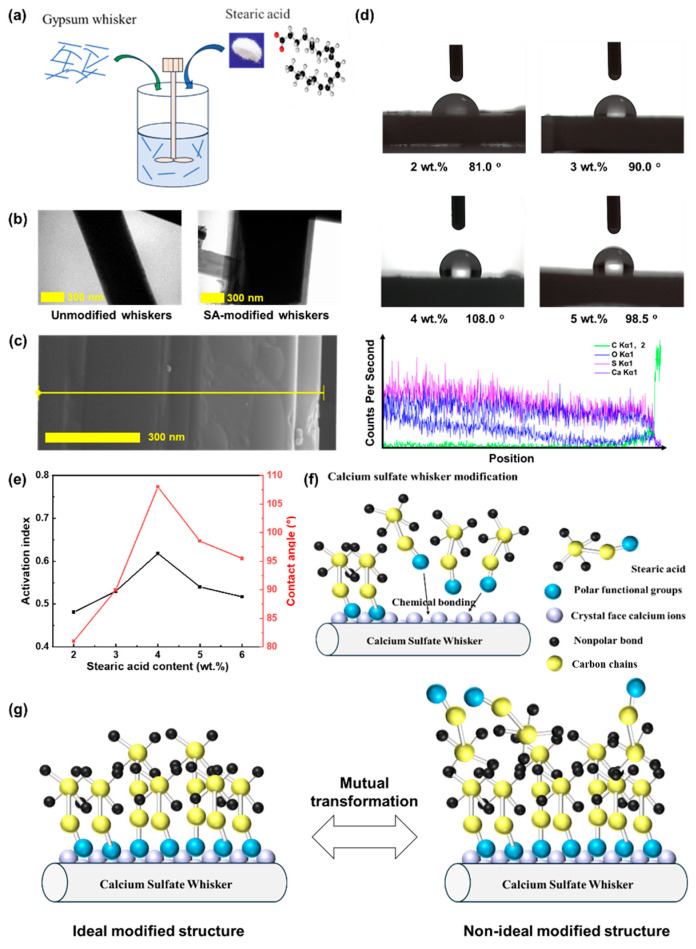
Surface modification of CSWs by SA. (**a**) Schematic of the modification process; (**b**) TEM images; (**c**) SEM-EDS line-scanning of SA-modified CSWs; (**d**) Contact angle of SA-modified CSWs; (**e**) Effect of SA content on the contact angle and activation index of CSWs; (**f**) Schematic of the modification mechanism; (**g**) Transformation of the modification mechanism.

Figure 2 illustrates the surface modification of CSWs by SA, confirming the feasibility of modifying CSWs with an SA/ethanol solution. The TEM images (Figure 2b) and SEM-EDS line-scanning image (Figure 2c) reveal the presence of a thin film coating the whisker surfaces. Additionally, as the SA content increases, both the activation index and contact angle initially rise and then decrease (Figure 2d,e). The activation index and contact angle reach their maximum values of 0.618 and 108°, respectively, when the SA content is 4 wt.%. Below 4 wt.% SA, incomplete surface coverage leads to insufficient hydrophobicity, limiting both the activation index and contact angle. In contrast, when the SA content exceeds 4 wt.%, excess SA promotes the intertwining of the modifier’s long hydrocarbon chains, causing hydrophilic groups to be exposed on the surface (Figure 2f,g). This transformation reverses the surface properties from hydrophobic to hydrophilic, thereby reducing both the activation index and contact angle. Consequently, 4 wt.% SA content is identified as the optimal modification concentration in this study. The SA content adsorbed on CSWs was determined by heating the modified whiskers at 150 °C on oil-absorbent paper for 4 h, replacing the paper hourly. Mass loss was monitored until stabilization, indicating complete SA removal. The adsorbed SA content was calculated to be 0.2 wt.% for CSWs modified with 4 wt.% SA.

**Figure 3:**
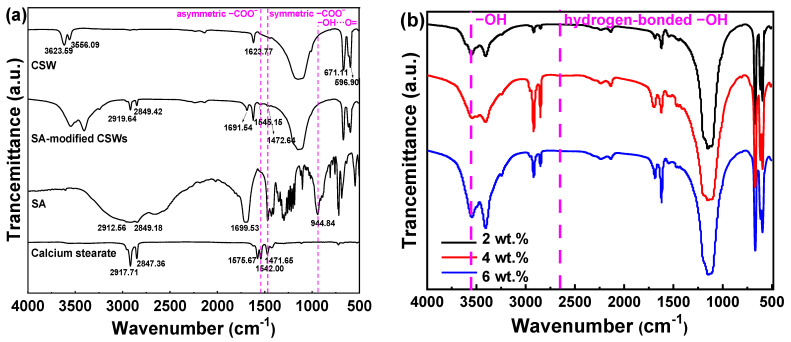
FT-IR spectra. (**a**) Unmodified CSWs, SA-modified CSWs, pure SA, and calcium stearate; (**b**) CSWs modified with varied SA concentrations.

Figure 3 shows the FT-IR spectra of unmodified CSWs, pure SA, calcium stearate, and CSWs modified with different contents of SA. The characteristic peaks at 2912.56 cm^−1^ and 2849.18 cm^−1^ are assigned to the stretching vibrations of –CH_3_ and –CH_2_ groups, respectively. The –OH stretching vibrations are observed at 3603.87 cm^−1^, representing the free hydroxyl groups in SA monomers. The peak at 2662.56 cm^−1^ is associated with hydrogen-bonded –OH groups in SA dimers. The –C=O stretching vibration appears at 1709.51 cm^−1^, while asymmetric and symmetric stretching vibrations of carboxylate groups (–COO^−^) are found in the range of 1469.48–1427.23 cm^−1^. The bending vibrations of –CH_2_ are located between 1350.94–1187.20 cm^−1^. The out-of-plane deformation vibration of the –OH···O= group in SA dimers is identified at 944.84 cm^−1^, and sulfate stretching vibrations appear in the 500–700 cm^−1^ region [24,25,26,27].

Based on these spectral features, unmodified CSWs exhibit the gypsum phase, while SA-related functional groups are identified in the spectra of pure SA, calcium stearate, CSWs modified with different contents of SA (Figure 3a,b). In particular, calcium stearate and CSWs modified with different contents of SA exhibit distinct double peaks near 1540 cm^−1^ (asymmetric -COO^−^ stretching) and around 1450 cm^−1^ (symmetric -COO^−^ stretching), which confirms that the formation of ionic bonds between the carboxyl groups of SA and Ca^2+^ ions [28]. Furthermore, as the SA content increases, the intensities of -CH_3_, -CH_2_-, and -C=O absorption bands also increase. The -OH stretching at 3603.87 cm^−1^ initially increases and then decreases, while the absorption at 2662.56 cm^−1^ continues to increase, indicating a rise in the formation of SA dimers. This observation supports the hypothesis that when the SA content exceeds 4 wt.%, the surface modification mechanism of CSWs gradually shifts from chemical bonding to physical adsorption. This transition contributes to the observed decrease in both the activation index and contact angle of the modified CSWs at higher SA contents.

**Figure 4:**
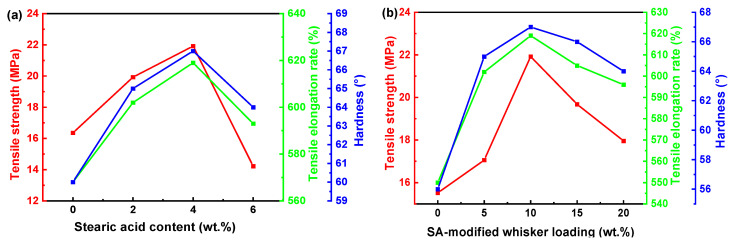
Mechanical properties of rubber composites. The red curve reflects tensile strength, the green curve reflects tensile elongation rate, and the blue curve reflects hardness. (**a**) varied SA content (10 wt.% whisker loading); (**b**) varied SA-modified CSWs loading (4 wt.% SA).

Figure 4 shows the Shore A hardness, tensile strength, and elongation at break of rubber composites containing CSWs modified with varied SA contents (at a fixed loading of 10 wt.%) and varied whisker loadings modified with 4 wt.% SA. The pure rubber exhibits a tensile strength of 15.52 MPa, an elongation at break of 550%, and a hardness of 56°. The addition of unmodified CSWs results in slight improvements, with values increasing to 16.36 MPa, 568%, and 60°, respectively. With the increase in SA content and whisker loading, mechanical properties of rubber composites exhibit an initial improvement followed by a subsequent decrease. The maximum values are achieved at 4 wt.% SA content and 10 wt.% whisker loading, where the tensile strength, elongation at break, and hardness reach 21.92 MPa, 619%, and 67°, respectively. Compared to the pure rubber, these represent increases of ~35%, ~8%, and ~10%, respectively. This shows that whisker additives significantly improve the mechanical properties of rubber.

**Figure 5:**
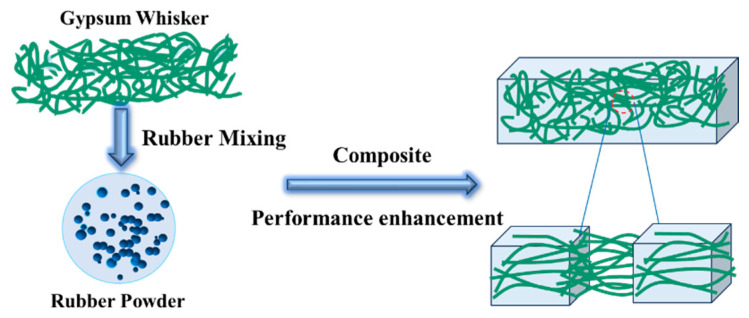
Schematic of the effect of SA-modified CSWs on the mechanical properties of rubber composites.

The mechanisms underlying the improvement in the mechanical properties of rubber were investigated. One contributing factor is the formation of CSWs can form a randomly oriented, misaligned, and spatially entangled network within the rubber matrix, enhancing its mechanical properties. In addition, surface modification with SA improves the interfacial compatibility between CSWs and the rubber. The long hydrocarbon chains of the SA modifier interact with the rubber matrix, strengthening the interfacial adhesion and enhancing the pinning effect between whiskers and polymer chains. However, excessive SA content can lead to an overly thick modification layer on the whisker surface, which hinders effective stress transfer and reduces reinforcement efficiency, as shown in Figure 5. Overall, chemical bonding through SA modification is more effective than physical adsorption in improving the mechanical performance of rubber composites.

## 4. Conclusions

(1) SA effectively modifies CSWs. As the SA content increases, the activation index and contact angle of the modified whiskers first rise and then decline. The maximum values of activation index (0.636) and contact angle (110°) are achieved when the SA content is 4 wt.%. Both chemical bonding and physical adsorption occur between SA and the whisker surface. With increasing SA content, the extend of chemical adsorption initially increases to a peak and then decreases, while physical adsorption continues to increase.
(2) Both unmodified and modified CSWs enhance the mechanical performance of rubber composites. The optimal mechanical properties are obtained at 4 wt.% SA content, yielding a hardness of 67°, a tensile strength of 21.92 MPa, and an elongation at break of 619%.

## Data Availability

The original contributions presented in this study are included in the article. Further inquiries can be directed to the corresponding author.

## References

[1] Nguyen H.A., Chen C.T., Chang T.P., Shih J.Y. (2023). Utilizations of preheated flue gas desulfurization gypsum and sulfate compositions to modify performances of super-sulfated cement. J. Therm. Anal. Calorim..

[2] Fan J., Wu F., Li D. (2018). Dynamic compressive response of a dendrite-reinforced Ti-based bulk metallic glass composite Author links open overlay panel. Mater. Sci. Eng. A.

[3] Guo J., Liu B., Zhang K., Sun Z., Mo E., Wang S., Liu J., Li Y., Xu L., Zhao Y. (2025). Long-term effects of a one-time application of flue gas desulfurization gypsum on the soil pore structure in sodic paddy fields. Agric. Water Manag..

[4] Du S.M., Long B., Mei Z.Y., Li Y.S., Li S., Wu X.Q., Chi R.A., Tan Y.Z., Li D.S. (2025). Toward zero-waste phosphogypsum valorization: Reengineered reverse-direct flotation synchronizes gypsum purification and functional co-products production. Chem. Eng. J..

[5] Zhou Y., Fang K., Chen Y., Chen Y., Li C., Chen Q. (2025). Reverse flotation purification of phosphogypsum and preparation of high whiteness CaSO_4_. Colloids Surf. A Physicochem. Eng. Asp..

[6] Lei Y., Gong Y.-J., He M., Li L., Qin J., Liu Y. (2024). High-Efficiency Purification and Morphology Regulation of CaSO_4_·2H_2_O Crystals from Phosphogypsum. Molecules.

[7] Aakriti, Bakshi P., Maiti S., Jain N. (2025). Hybrid composite binder development using flue gas desulfurization gypsum and ground granulated blast furnace slag: Characterization and life cycle assessment. J. Indian Chem. Soc..

[8] Choi C.Y. (2025). Hydration and Mechanical Properties of Low-Carbon Binders Using CFBC Ash. Materials.

[9] Bakshi P., Pappu A., Bharti K.D. (2025). Life cycle assessment for calcination process of flue gas desulfurization gypsum and transformation into β-CaSO_4_·0.5H_2_O. Sustain. Chem. Environ..

[10] Sina T.A., Brahim A.J., Ali B.B., Achiou B., Haneklaus N., Beniazza R. (2024). Securing gypsum demand in cement industry by gypsum by-products: Current challenges and prospects. Mater. Today Sustain..

[11] Lun W.L., Lin Y.W., Feng Z., Zhou Q.S., Liu G.H., Peng Z.H., Qi T.G., Shen L.T., Li X.B. (2024). Coal fly ash resource utilization: Effects of inorganic minerals amendments on CFA-originated opal/sand aggregates formation. J. Cent. South Univ..

[12] An K., Li S., Guan X. (2025). Influence of dicarboxylic acid modifiers on the growth habits of α-hemihydrate gypsum crystals prepared from desulfurized gypsum and its mechanisms of action. Constr. Build. Mater..

[13] Aakriti, Maiti S., Jain N., Prajapati P. (2024). Synthesis of calcium sulfate whiskers via acidification exploiting FGD gypsum for improved binder properties. Sustain. Chem. Pharm..

[14] Wang X., Jin B., Fan M., Liu X., Zhang X., Zhang J., Li S., Zhang W. (2023). A Feasible Route for Preparation of Calcium Sulfate Whiskers from FGD Gypsum via Filtrate Recycle under Hydro-Thermal Conditions. Processes.

[15] Wang X., Jin B., Xu Z., Zhang X., Liu X., Yang L. (2019). Effects of AlCl_3_ on the Crystal Morphology of Calcium Sulfate Whisker Prepared from FGD Gypsum. IOP Conf. Ser. Mater. Sci. Eng..

[16] Ji X.W., Lu Z., Wang J., Ye N., Zhang H., Zhou L., Li J., Lu Y. (2024). Construction of micro-nano hybrid structure based on carbon nanotube whisker and alumina for thermally conductive yet electrically insulating silicone rubber composites. Compos. Sci. Technol..

[17] Silva J.M., Dias J.Y., Zaszczyńska A., Kołbuk D., Kowalczyk T., Sajkiewicz P.Ł., Yarin A.L. (2023). Three-phase bio-nanocomposite natural-rubber-based microfibers reinforced with cellulose nanowhiskers and 45S5 bioglass obtained by solution blow spinning. J. Appl. Polym. Sci..

[18] Wu X., Lu C., Han Y., Zhou Z., Yuan G., Zhang X. (2016). Cellulose nanowhisker modulated 3D hierarchical conductive structure of carbon black/natural rubber nanocomposites for liquid and strain sensing application. Compos. Sci. Technol..

[19] Chen Y., Ding Y., Dong Y., Liu Y., Ren X., Wang B., Gao C. (2020). Surface modification of calcium sulfate whisker using thiol-ene click reaction and its application in reinforced silicone rubber. J. Polym. Sci..

[20] Li D., Zhao H., Jia Z., Jia D. (2025). Preparation of phthalonitrile resin/calcium sulfate whisker composites with enhanced wear and heat resistance. High Perform. Polym..

[21] Shi P., Deng Z., Yuan Y., Sun J. (2010). Preparation of Calcium Sulfate Whiskers from Desulphurized Gypsum by Hydrothermal Synthesis. J. Northeast. Univ. (Nat. Sci.).

[22] (1999). State Bureau of Quality and Technical Supervision of China. Rubber—Determination of Indentation Hardness by Means of Pocket Hardness Meters.

[23] (1998). State Bureau of Quality and Technical Supervision of China. Rubber, Vulcanized or Thermoplastic—Determination of Tensile Stress-Strain Properties.

[24] Baeten J., Romanus K., Degryse P., De Clercq W. (2009). Application of a multi-analytical toolset to a 16th century ointment: Identification as lead plaster mixed with beeswax. Microchem. J..

[25] Silva D.A.E., Caseli L., Olivati A.D.C. (2017). Organization of polythiophenes at ultrathin films mixed with stearic acid investigated with polarization-modulation infrared reflection–absorption spectroscopy. Colloids Surf. A Physicochem. Eng. Asp..

[26] Das E., Mustard J.F., Tarnas J.D., Pascuzzo A.C., Kremer C.H. (2022). Investigating the origin of gypsum in Olympia Undae: Characterizing the mineralogy of the basal unit. Icarus.

[27] Yakovlev G., Gordina A., Drochytka R., Buryanov A.F., Smirnova O. (2020). Structure and properties of modified gypsum binder. Smart Sustain. Built Environ..

[28] Schrank S., Kann B., Saurugger E., Hainschitz M., Windbergs M., Glasser B.J., Khinast J., Roblegg E. (2015). The effect of the drying temperature on the properties of wet-extruded calcium stearate pellets: Pellet microstructure, drug distribution, solid state and drug dissolution. Int. J. Pharm..

